# The Efficacy and Outcomes of Renal Replacement Therapy in Pediatric Metabolic Disorders

**DOI:** 10.3390/jcm13216452

**Published:** 2024-10-28

**Authors:** Hülya Gözde Önal, Hülya Nalçacıoğlu, Işıl Özer, Demet Tekcan Karalı

**Affiliations:** 1 Department of Pediatric Nephrology, Faculty of Medicine, Ondokuz Mayıs University, 55270 Samsun, Turkey; hulyanalcacoglu@hotmail.com (H.N.); demettekcan@yahoo.com (D.T.K.); 2 Department of Pediatric Metabolism, Faculty of Medicine, Ondokuz Mayıs University, 55270 Samsun, Turkey; isil.ozer@omu.edu.tr

**Keywords:** renal replacement therapy, inborn errors of metabolism, maple syrup urine disease, methylmalonic acidemia, glycogen storage disease

## Abstract

**Background/Objectives:** This study aims to evaluate the efficacy and outcomes of renal replacement therapy (RRT) in pediatric patients with metabolic diseases, specifically focusing on the impact of hemodialysis (HD) and peritoneal dialysis (PD) on clinical parameters, toxin reduction, and long-term survival. **Methods:** This retrospective study included 10 pediatric patients (eight females and two males) treated at a pediatric nephrology department between 2020 and 2023. Patients diagnosed with metabolic disorders, including maple syrup urine disease (MSUD), methylmalonic acidemia (MMA), and glycogen storage disease (GSD), underwent RRT. Clinical data, demographic information, and biochemical parameters were collected and analyzed. **Results:** Among the patients, 50% were diagnosed with MSUD, 30% with MMA, and 20% with GSD. RRT, including HD and PD, was administered to manage acute metabolic crises. HD was particularly effective in rapidly reducing toxic metabolite levels. Patients treated with HD showed significant reductions in leucine and ammonium levels, with median reductions of 94.5% and 86%, respectively. Overall, 60% of the patients demonstrated long-term survival, highlighting the critical role of RRT in managing metabolic crises. In conclusion, RRT, including HD and PD, is crucial in managing pediatric metabolic disorders by effectively reducing toxic metabolite levels and improving clinical outcomes. **Conclusions:** The results of this study are consistent with previous research, highlighting the critical role of RRT in the acute management of metabolic crises and supporting its adoption as a standard treatment method.

## 1. Introduction

Pediatric metabolic disorders are complex diseases arising from congenital genetic abnormalities that affect specific biochemical pathways in the body. These disorders are characterized by the obstruction of metabolic pathways and the accumulation of toxic substances due to enzyme deficiencies or dysfunctions. Organic acidemias (OAs), maple syrup urine disease (MSUD), and other congenital metabolic diseases can present severe clinical conditions early in life, requiring urgent medical intervention [1,2,3,4].

Organic acidemias result from enzyme deficiencies that lead to the accumulation of specific organic acids in the body. This accumulation can cause ketoacidosis, hyperammonemia, and other metabolic disturbances [5,6]. Similarly, MSUD arises from a defect in the metabolism of branched-chain amino acids, leading to the accumulation of toxic metabolites like leucine, which has neurotoxic effects. These conditions can result in severe neurological complications such as brain edema, coma, and death [7,8].

In addition to organic acidemias, glycogen storage disease (GSD) type 1 is another congenital metabolic disorder that can lead to severe clinical manifestations. GSD type 1 results from enzyme deficiencies that disrupt glycogen metabolism, leading to the accumulation of glycogen in organs such as the liver and kidneys. This can cause serious complications like hypoglycemia, lactic acidosis, and renal failure. In such cases, renal replacement therapy (RRT) is crucial for managing lactic acidosis and acute renal failure, helping to stabilize patients during metabolic crises [9].

In managing congenital metabolic diseases, RRT is critically important for rapidly removing toxic metabolites. RRT can be life-saving during metabolic crises. Modalities of RRT, such as hemodialysis (HD) and peritoneal dialysis (PD), can be used in different clinical situations, each with its unique advantages and limitations. Previous studies have shown that RRT is effectively used in hyperammonemia and other metabolic emergencies [10,11,12].

This study aims to evaluate the efficacy and outcomes of RRT in patients with pediatric metabolic diseases.

## 2. Materials and Methods

### 2.1. Study Design and Patient Selection

This retrospective study included 10 pediatric patients (8 females, 2 males) referred for detoxification to the Pediatric Nephrology Department of Samsun Ondokuz Mayıs University Faculty of Medicine between 2020 and 2023. Ethical approval for this study was obtained from the Clinical Research Ethics Committee of Samsun Ondokuz Mayıs University (approval number: KAEK 2023/272, dated 23 January 2024). All patients were monitored with a diagnosis of metabolic disease. Demographic data, medical histories, clinical findings, and biochemical parameters of the patients were thoroughly examined. The Metabolic Diseases Department confirmed the diagnoses of metabolic diseases, and subsequently, dialysis treatment was administered in the Pediatric Nephrology Department. The modalities of dialysis and vascular access methods were tailored to the individual clinical conditions of each patient.

### 2.2. Pre-Dialysis Medical Treatment

Before dialysis, medical treatments were administered to stabilize the patients and reduce toxin levels. Patients with hyperammonemia were given intravenous (IV) sodium benzoate, phenylacetate (Ammonul), L-arginine, and carbamyl glutamate. Patients with organic acidemia were treated with hydroxocobalamin, carnitine, and biotin. Additionally, all patients received glucose infusions devoid of protein to provide energy. Treatment protocols were tailored to each patient’s metabolic disorder and clinical condition.

In all cases, protein intake was immediately discontinued. Patients were given high-dose glucose infusions at 10–12 mg/kg/day and lipid infusions at 2 g/kg/day. For those with blood glucose levels over 250 mg/dL, insulin infusions were started at 0.05–0.1 U/kg/h and continued until blood glucose levels dropped below 180 mg/dL. All hyperammonemic patients received carglumic acid (loading dose: 250 mg/kg, maintenance: 100 mg/kg/day, orally), carnitine (100–200 mg/kg/day, orally), and sodium benzoate (250 mg/kg/day, orally). Protein intake was resumed within two days.

For patients with OA, amino acid mixtures lacking precursor amino acids such as threonine, methionine, valine, and isoleucine were utilized (Milupa OS1; Nutricia, Amsterdam, Netherlands). Patients diagnosed with methylmalonic acidemia (MMA) were treated with hydroxocobalamin at a dose of 1 mg/day via intramuscular injection. For patients with MSUD, enteral feeding consisted of mixtures free of branched-chain amino acids (leucine, isoleucine, and valine) (Milupa MSUD1; Nutricia), along with thiamine supplementation at 10 mg/day orally as a cofactor for enzyme activity.

### 2.3. Dialysis Modalities and Implementation Protocols

Hemodialysis (HD) and peritoneal dialysis (PD) have distinct advantages and limitations, especially in cases of acute metabolic crises. Hemodialysis can be applied immediately because of its ability to establish vascular access rapidly and its high efficiency in removing toxins within a short time frame. This makes it the preferred option in urgent situations where rapidly reducing metabolite levels, such as leucine in MSUD or ammonium in MMA, is critical. On the other hand, PD requires a more gradual initiation, especially post-surgical catheter placement, with incremental increases in fluid volumes. This gradual process makes PD more suitable for stable patients or neonates where a slower toxin clearance may be safer.

#### 2.3.1. Hemodialysis

RRT is considered the primary treatment for the rapid removal of high levels of toxic metabolites. The RRT methods used include hemodialysis and PD. For HD, dual-lumen French catheters suitable for each patient’s age and weight were inserted into either the femoral or subclavian veins. The dialysis circuit was pre-prepared using heated packed red blood cells and a saline or normal saline solution containing 5% albumin. Based on the patient’s coagulation status, a heparin bolus of 15 IU/kg was administered, followed by a continuous infusion of 15–20 IU/kg/h. The blood flow rate was maintained at 150–300 mL/min, and the dialysate flow rate was set between 300 and 500 mL/min. Fluid loss was adjusted to achieve a slightly positive fluid balance. HD procedures were performed using Shunmei brand acute HD catheters. Fresenius FX40 CorDiax dialyzers(Manufactured by Fresenius Medical Care, Bad Homburg, Germany), Gambro AK 96(Manufactured by Baxter International, Deerfield, Illinois, USA), and Gambro AK 98 hemodialysis machines(Manufactured by Baxter International, Deerfield, Illinois, USA) were utilized during HD. To reduce the risk of hypoglycemia, all patients received an intravenous (IV) 10% glucose infusion during dialysis. HD sessions lasted 4–6 h, depending on each patient’s clinical condition.

#### 2.3.2. Peritoneal Dialysis

PD was used in neonatal patients. Peritoneal dialysis was carried out using an Automated Peritoneal Dialysis device (Home-Choice Baxter, Manufactured by Baxter International, Deerfield, IL, USA) with a 32 cm swan-neck neonatal catheter. Initial fill volumes were 20–30 mL/kg, with dwell times ranging between 15 and 20 min. The PD solutions used were standard with 1.36% or 2.27% glucose concentrations. Lactate-buffered standard solutions were used as the PD solution. Ceftazidime was administered for intraperitoneal antibiotic therapy.

### 2.4. Vascular Access

For HD, dual-lumen French catheters appropriate for each patient’s age and weight were inserted into the femoral or subclavian veins. These catheters are capable of withstanding high blood flow rates. In neonatal patients for PD, swan-necked neonatal catheters (32 cm) were placed under intravenous sedation. These catheters were appropriately positioned in the peritoneal cavity for optimal fill volumes and dwell times.

### 2.5. Analysis

Statistical analyses were conducted using SPSS for Windows (Version 29, Chicago, IL, USA). Descriptive statistics were utilized to summarize the data, with categorical variables presented as counts and percentages and continuous variables as medians [IQR 25th–75th]. The normality of the data distribution was assessed using the Shapiro–Wilk test and histograms. Group comparisons for categorical variables were performed using the Pearson chi-square test or Fisher’s exact test when expected frequencies were below 5. For continuous variables, the Mann–Whitney U test was employed because of the non-normal distribution of the data. Statistical significance was set at a two-sided *p*-value of <0.05. Additionally, this study assessed toxin level reductions pre- and post-dialysis, focusing on changes in leucine and ammonium levels, which were depicted through descriptive statistics and graphical methods. ROC curves were generated to assess the diagnostic accuracy of the parameters, and sensitivity, specificity, positive likelihood ratios, and negative likelihood ratios were calculated based on the optimal cut-off values determined by the maximum Youden index.

## 3. Results

A total of ten patients were enrolled in this study, with 80% being female (*n* = 8) and 20% male (*n* = 2) (Table 1). The etiological breakdown revealed that 50% (*n* = 5) of the patients were diagnosed with maple syrup urine disease (MSUD), 30% (*n* = 3) with methylmalonic acidemia (MMA), and 20% (*n* = 2) with glycogen storage disease (GSD). Of the two patients with GSD, one had glycogen storage disease type 1, and the other had Fanconi–Bickel syndrome. The median age of the patients was 637.5 days (IQR 3.75–4148.75), and the median weight was 9.75 kg (IQR 3.23–18). Long-term survival was observed in 60% of the patients (*n* = 6). Table 2 provides detailed information on each patient’s gender, etiology, weight, type of dialysis, the initiation day of dialysis during the hospital stay, the indication for dialysis, the age, and the number of dialysis sessions undertaken.

The study group was divided into the following two categories based on the type of dialysis received: hemodialysis (60%, *n* = 6) and peritoneal dialysis (40%, *n* = 4). The median age in the hemodialysis group was 3617.5 days (IQR 728.75–4873.85 days), which was significantly higher than the median age of 3.5 days (IQR 2.25–8.5 days) in the peritoneal dialysis group (*p* = 0.01). The gender distribution within the dialysis groups showed that 66.7% (*n* = 4) of the hemodialysis patients were female, compared with 100% (*n* = 4) in the peritoneal dialysis group. Additionally, the median weight in the hemodialysis group was 16 kg (IQR 10.88–27.75 kg), significantly higher than the median weight of 2.95 kg (IQR 1.73–4.03 kg) in the peritoneal dialysis group (*p* = 0.01) (Table 3).

**Figure 1:**
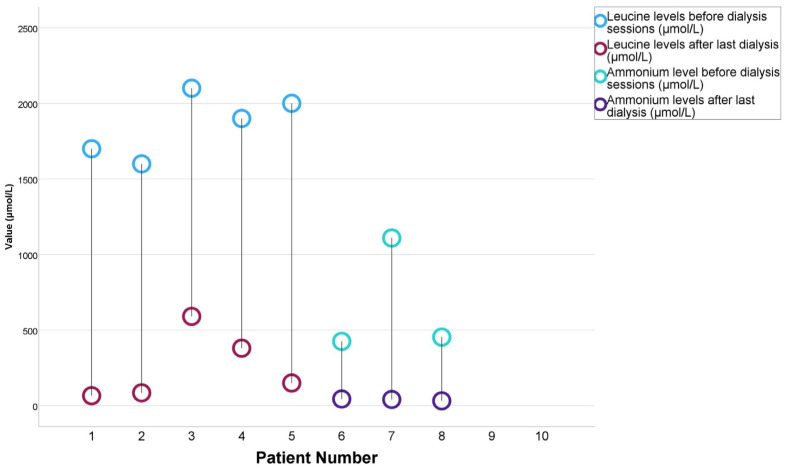
Changes in leucine and ammonium levels before and after dialysis sessions in the study patients.

In terms of etiology, the hemodialysis group comprised 16.7% (*n* = 1) of the patients with MSUD, 50% (*n* = 3) with MMA, and 33.3% (*n* = 2) with GSD. Conversely, in the peritoneal dialysis group, all patients (100%, *n* = 4) were diagnosed with MSUD. The median day for initiating dialysis post-admission was 2 days (IQR 1–3 days) in the hemodialysis group, compared to 3.5 days (IQR 2.25–8.5 days) in the peritoneal dialysis group, with no statistically significant difference between the groups (*p* = 0.114). The median number of dialysis sessions was 3.5 (IQR 2.75–5.75) in the hemodialysis group and 6.5 (IQR 3–11.5) in the peritoneal dialysis group, also showing no significant difference (*p* = 0.476). The median toxin reduction achieved was 94.5% (IQR 90.4–96.2 %) in the hemodialysis group and 86% (IQR 73.3–93.5 %) in the peritoneal dialysis group, with the difference not reaching statistical significance (*p* = 0.200) (Table 3). Changes in leucine and ammonium levels before and after dialysis sessions for individual patients are depicted in Figure 1.

## 4. Discussion

The objective of this study was to assess the efficacy and outcomes of RRT in managing pediatric metabolic disorders. The study results showed that HD and PD effectively treated pediatric patients with metabolic diseases requiring RRT. It was observed that patients undergoing HD had higher demographic factors such as age and weight compared with those receiving PD. However, there was no statistically significant difference between the two groups in terms of toxin reduction rates and long-term survival rates. These findings suggest that HD and PD are effective therapeutic modalities that can be used to manage pediatric metabolic disorders.

MSUD is an autosomal recessive disorder caused by a defect in the metabolism of branched-chain amino acids (leucine, isoleucine, and valine). This metabolic disorder results from a deficiency in the function of the branched-chain alpha-keto acid dehydrogenase complex, leading to the accumulation of these amino acids at toxic levels. The pathophysiology of MSUD is characterized by the neurotoxic effects of leucine accumulation. Leucine can cause cerebral edema, astrocyte swelling, and myelin damage, leading to severe neurological complications, coma, and even death. Significant clinical findings of MSUD include feeding difficulties, vomiting, lethargy, changes in muscle tone (either increased or decreased), seizures, and the characteristic sweet, maple syrup odor of the urine. RRT is vital in the acute management of MSUD for the rapid clearance of toxins and prevention of neurological damage. This treatment modality plays a critical role in swiftly reducing high toxin levels and improving the overall clinical condition of patients [13,14,15]. Five patients diagnosed with MSUD and treated with dialysis were included in this study, and significant decreases in leucine levels were achieved in all cases. RRT was used effectively to rapidly lower toxin levels in these patients.

MMA is an autosomal recessive metabolic disorder resulting from a deficiency of the enzyme methylmalonyl-CoA mutase or a lack of its cofactor, adenosyl-cobalamin (vitamin B12). This disease is closely related to propionic acidemia, and in both conditions, impaired propionic acid metabolism results in the accumulation of toxic metabolites. The pathophysiology of MMA involves the accumulation of methylmalonic acid and other toxic metabolites, leading to metabolic acidosis, hypoglycemia, hyperammonemia, and organ dysfunction. Significant clinical findings include vomiting, lethargy, hypotonia, seizures, developmental delay, and recurrent metabolic crises. These crises can result in severe neurological and cardiac complications [16,17,18]. In our study, three patients were diagnosed with MMA and underwent RRT, and significant reductions in ammonia levels were observed during this treatment. The marked decrease in ammonia levels before and after dialysis therapy confirms the critical role of RRT in the management of MMA and its high efficacy in clearing toxic metabolites.

GSD is an inherited disorder resulting from deficiencies or dysfunctions of enzymes involved in glycogen metabolism. This disease is characterized by excessive glycogen accumulation in the liver, muscles, and other tissues, leading to various clinical manifestations such as hypoglycemia, lactic acidosis, and organomegaly. The pathophysiology of GSD involves the improper breakdown or synthesis of glycogen, which disrupts energy metabolism and causes structural damage to the affected organs. Significant clinical findings of GSD include muscle weakness, growth retardation, hypoglycemic episodes, and hepatomegaly [19,20,21]. In this study, two patients were diagnosed with GSD and underwent RRT, and significant improvements were observed during the treatment process. The metabolic imbalances caused by GSD were effectively managed with RRT, resulting in notable improvements in the overall clinical condition of the patients.

The importance of RRT in metabolic inborn errors is well-known. Various studies have highlighted the positive effects of this treatment modality on clinical outcomes in patients. In the study by Porta et al., the overall survival rate of neonates undergoing emergency RRT because of congenital metabolic diseases was reported to be 58.3%. Their study showed that hyperammonemia neonates responded rapidly to RRT, with ammonia levels dropping below 300 µmol/L within 8 h [22]. Similarly, in the study by Eisenstein et al., the efficacy of HD in 20 neonates with congenital metabolic disorders was evaluated. Their study found that HD reduced leucine levels by 92.1% in patients with MSUD and ammonia levels by 86.5% in patients with hyperammonemia. This treatment modality led to significant clinical improvements in 85% of the patients; however, three patients died in the neonatal period, and four patients died during long-term follow-up [23]. In the study by Celik et al., the efficacy of continuous venovenous hemodiafiltration (CVVHDF) and PD were compared in 40 neonates with congenital metabolic disorders. Their study reported that CVVHDF facilitated faster elimination of toxic metabolites from the body and reduced ammonia levels more quickly than PD. However, the two dialysis modalities had no significant difference in mortality rates [24]. Our study yielded similar findings. RRT applied to children with metabolic disorders played a crucial role in rapidly removing toxic metabolites and restoring metabolic balance. The findings in the literature, consistent with the results of our study, demonstrate the critical importance of RRT in managing metabolic inborn errors.

While this study focuses on the clinical application of RRT in metabolic disorders, the comparison of hemodialysis and peritoneal dialysis reveals important distinctions. Hemodialysis offers a rapid solution for life-threatening metabolic crises, especially in patients with severe hyperammonemia or elevated leucine levels. Peritoneal dialysis, while less immediate in its effects, provides a gentler, sustained clearance of toxins, particularly in neonates or those with contraindications for aggressive fluid shifts. The selection of dialysis modality should, therefore, be guided by both the urgency of the clinical situation and patient-specific factors such as age, weight, and the severity of the metabolic crisis.

While peritoneal dialysis is not the preferred method for treating metabolic diseases, it was used in this study because of the lack of availability of hemodiafiltration. The timing of the decision to initiate dialysis after the onset of metabolic crises played a crucial role in stabilizing patients, with faster intervention leading to better outcomes. Additionally, the duration and recurrence of dialysis sessions were critical in maintaining metabolic balance, especially in cases of severe metabolic crises [25,26].

Given the well-established role of RRT in the management of metabolic disorders, future studies could benefit from a systematic review of the existing literature to further explore the nuanced differences between hemodialysis and peritoneal dialysis. Specific criteria, such as clinical urgency and individual patient factors, should be analyzed to develop more comprehensive guidelines for modality selection in pediatric metabolic crises. Our current findings provide a basis for such an exploration.

## 5. Limitations

This study has several limitations. Firstly, the limited number of patients and the fact that it was conducted at a single center may hinder the generalizability of the results. Additionally, being a retrospective study, there may be limitations regarding the accuracy and completeness of the data. When comparing the efficacy of different dialysis modalities, the effects of factors such as patients’ clinical status, disease severity, and other treatment protocols could not be fully controlled. In the future, these findings must be validated through larger patient groups and multicenter prospective studies.

## 6. Conclusions

This study examined the efficacy and outcomes of RRT in pediatric metabolic disorders. The findings demonstrated that HD and PD play a significant role in the rapid clearance of toxic metabolites and in restoring metabolic balance. These results suggest that RRT should be considered a standard treatment approach for pediatric metabolic disorders and highlight the importance of further research in this field.

## Figures and Tables

**Table 1:** Demographic and clinical characteristics of the study patients.

Characteristic		N (%)/Median (IQR)
**Sex**	*Female*	8 (80%)
	*Male*	2 (20%)
**Etiology**	*MSUD*	5 (50%)
	*MMA*	3 (30%)
	*GSD*	2 (20%)
**Age (days)**		637.5 (3.75–4148.75)
**Weight (kg)**		9.75 (3.23–18)
**Long-term Survival**		6 (60%)

**MSUD:** maple syrup urine disease, **MMA:** methylmalonic acidemia, **GSD:** glycogen storage disease.

**Table 2:** Individual patient characteristics and dialysis details.

Patients	Sex	Etiology	Weight (kg)	Dialysis Type	Dialysis Initiation Day	Dialysis Indication	Age at Dialysis (Days)	Number of Dialysis Sessions
P1	M	*MSUD*	48	HD	1	High leucine + Encephalopathy	5260	8
P2	F	*MSUD*	4.2	PD	10	Seizure + Encephalopathy	10	3
P3	F	*MSUD*	2.4	PD	3	High leucine + Kidney failure	3	12
P4	F	*MSUD*	1.51	PD	4	High leucine	4	10
P5	F	*MSUD*	3.5	PD	2	High leucine	2	3
P6	M	*MMA*	7.5	HD	2	Encephalopathy + Septic shock + Kidney failure	455	3
P7	F	*MMA*	17	HD	1	Seizure + Septic shock	820	4
P8	F	*MMA*	21	HD	3	Septic shock + Kidney failure	4745	2
P9	F	*GSD* (*T1*)	12	HD	3	Metabolic crisis	3950	3
P10	F	*GSD* (*FBS*)	15	HD	2	Metabolic crisis	3285	5

**MSUD:** maple syrup urine disease; **MMA:** methylmalonic acidemia; **GSD:** glycogen storage disease; **T1:** type 1; **FBS:** Fanconi–Bickel syndrome; **HD:** hemodialysis; **PD:** peritoneal dialysis.

**Table 3:** Comparison of the hemodialysis and peritoneal dialysis groups.

Characteristic	Hemodialysis (*n* = 6)	Peritoneal Dialysis (*n* = 4)	*p*
**Age (days), median (IQR)**	3617.5 (728.75–4873.75)	3.5 (2.25–8.5)	0.01
**Sex (female), N (%)**	4 (66.7%)	4 (100%)	
**Weight (kg), median (IQR)**	16 (10.88–27.75)	2.95 (1.73–4.03)	0.01
**Etiology, N (%)**			
**MSUD**	1 (16.7%)	4 (100%)	
**MMA**	3 (50%)	0 (0%)	
**GSD**	2 (33.3%)	0 (0%)	
**First dialysis day, median (IQR)**	2 (1–3)	3.5 (2.25–8.5)	0.114
**Number of dialysis sessions, median (IQR)**	3.5 (2.75–5.75)	6.5 (3–11.5)	0.476
**Toxin reduction (%), median (IQR)**	94.5 (90.4–96.2)	86 (73.3–93.5)	0.200

**MSUD:** maple syrup urine disease, **MMA:** methylmalonic acidemia, **GSD:** glycogen storage disease.

## Data Availability

The entire deidentified dataset, data dictionary and analytic code for this investigation are available upon request, from the date of article publication by contacting Hülya Gözde Önal, at hulyanalcacoglu@hotmail.com.
